# A Case of Cardiac Arrest Caused by Air Embolism from Routine Root Canal Procedure

**DOI:** 10.14797/mdcvj.1067

**Published:** 2022-09-20

**Authors:** Akarsh Parekh, Joel McCormick, Atheer Hussain-Amin, Bryan Barnosky, Matthew Edwards

**Affiliations:** 1 Department of Cardiovascular Medicine, McLaren Macomb Medical Center, Mount Clemens, Michigan, US; 2 Michigan State University, East Lansing, Michigan, US; 3 Department of Internal Medicine, McLaren Macomb Medical Center, Mount Clemens, Michigan, US; 4 Department of Critical Care Medicine, McLaren Macomb Medical Center, Mount Clemens, Michigan, US

**Keywords:** cardiopulmonary arrest, air embolism, root canal procedure, dental surgery

## Abstract

Venous air embolism (VAE) occurs when air is introduced into the venous system and subsequently travels into the right heart and pulmonary circulation. VAE mainly occurs from air that is forced by positive pressure or drawn in by negative pressure. We present a rare case of fatal VAE that occurred during a routine dental root canal procedure. A 69-year-old male was undergoing a root canal procedure at an outpatient dental office under local anesthesia. During the procedure, he went into cardiopulmonary arrest. He was resuscitated, and return of spontaneous circulation was achieved. Thoracic computed tomography was performed and revealed large amounts of air within the right ventricle and portal venous system. VAE should be recognized as a potentially fatal complication resulting from routine dental procedures.

## Introduction

Venous air embolism (VAE) occurs when air is introduced into the venous system and subsequently travels into the right heart and pulmonary circulation.^1^ VAE mainly occurs from air that is forced by positive pressure or drawn in by negative pressure.^2^ Small amounts of embolized air essentially remains undetected because it rarely leads to sequelae.^2^ When a patient is in upright positioning, their central venous pressure can drop as low as 20 to 25 mm Hg during the inspiratory cycle.^2^ When air gains access to the venous system, it flows through the right heart, subsequently obstructing the right ventricular outflow tract and/or pulmonary arteries.^2^ The factors that affect VAE sequelae depend on the volume of embolized air, the time it takes to enter the venous system, and the patient’s position.^1,2^ Studies have shown that 200 to 300 mL of air embolization can be fatal in humans.^3^ The strength of the right ventricle (RV) plays a role in the severity of complications that may arise from the VAE.^2^

When trapped in a beating RV, air and blood can be whipped into foam, further hindering flow through the right side of the heart.^2^ This can result in immediate death due to cerebral hypoxia, right ventricular failure due to severe dilation, or left ventricular dysfunction from hypoxia.^2^ VAE during endodontic therapy can occur when compressed air is used to dry the root canal.^4^ The introduction of pressured air during dental procedures can lead to subcutaneous emphysema if it disrupts intraoral barriers.^5^ Moreover, neurosurgical procedures performed on a patient in a sitting position have the highest incidence of VAE.^1^ We herein present a case of cardiac arrest from an air embolism in a patient undergoing routine dental treatment. We hypothesize that the pressurized air used during the root canal procedure introduced air into the central system through the pterygoid venous plexus, which is located in the gums.

## Case description

A 69-year-old man with a history of heart failure and hypertension presented to the hospital with pulseless electrical activity cardiac arrest while undergoing a routine root canal dental procedure. The patient had a past medical history of congestive heart failure with reduced ejection fraction, hypertension, diabetes mellitus type 2, and hyperlipidemia. In addition, he had a past surgical history of intracardiac defibrillator along with no significant social history. He had no known drug allergies and takes losartan, carvedilol, furosemide, and atorvastatin on a daily basis. He was undergoing a root canal procedure at an outpatient dental office under local anesthesia. During the procedure, he was found to be unresponsive, which prompted the office to call emergency medical services. Subsequently, he was found to be in pulseless electrical activity, and cardiopulmonary resuscitation was initiated. He was resuscitated, and return of spontaneous circulation was achieved upon arrival to the emergency department. His total downtime was 25 minutes.

**Figure 1:**
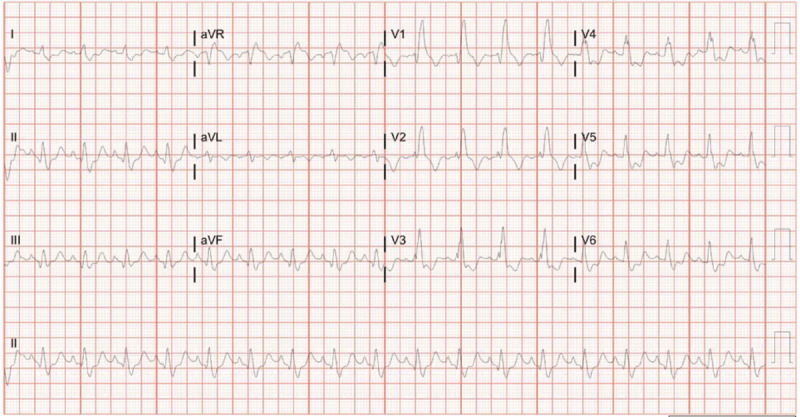
Electrocardiogram.

**Figure 2:**
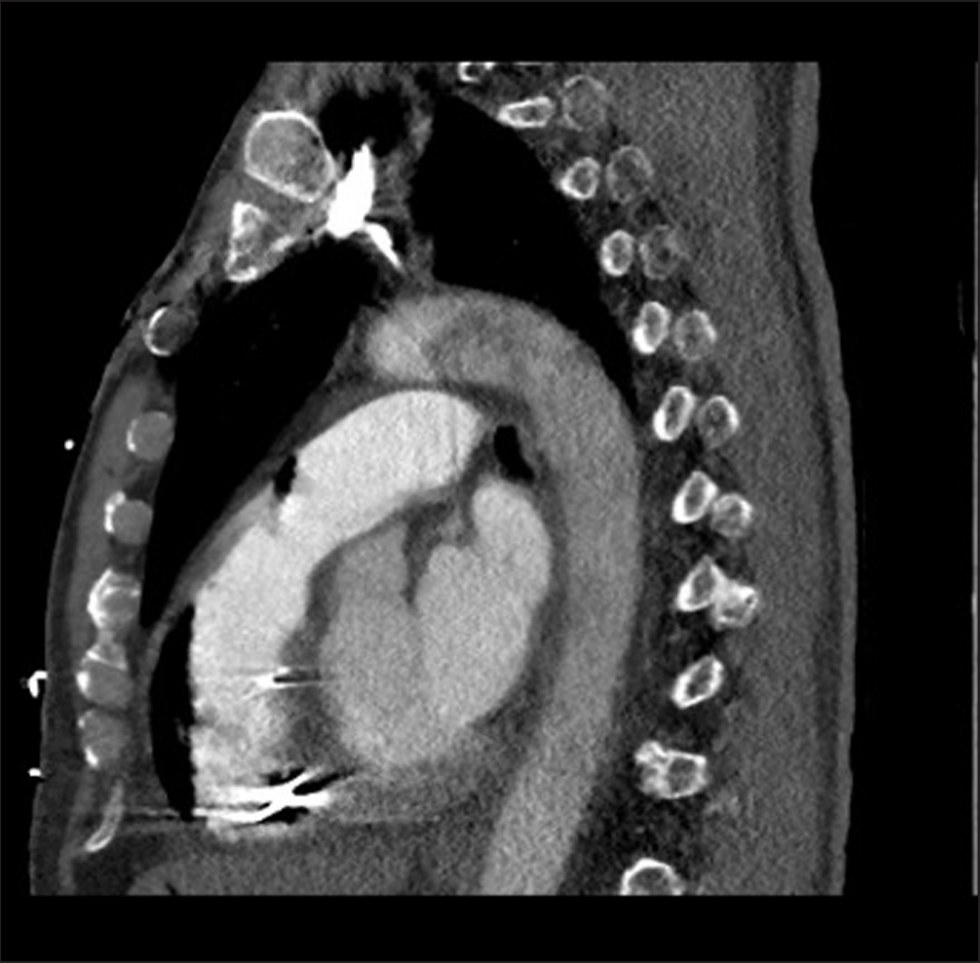
Computed tomography sagittal view demonstrating right ventricular air embolism with extension into the pulmonary trunk.

**Figure 3:**
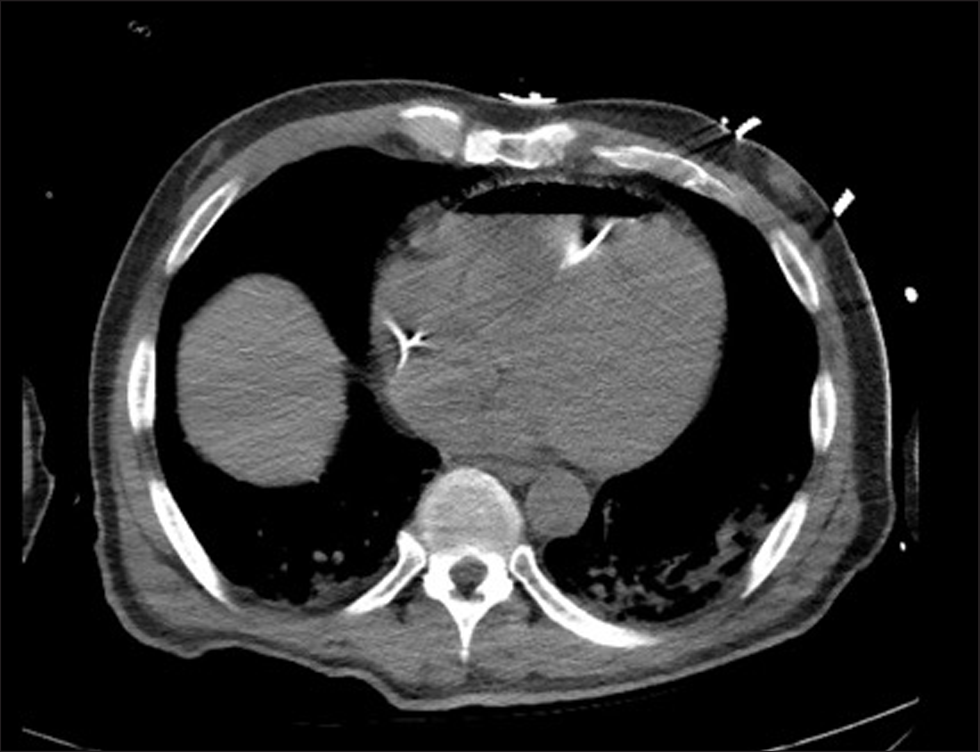
Computed tomography chest axial view showing presence of air in the right ventricle.

**Figure 4:**
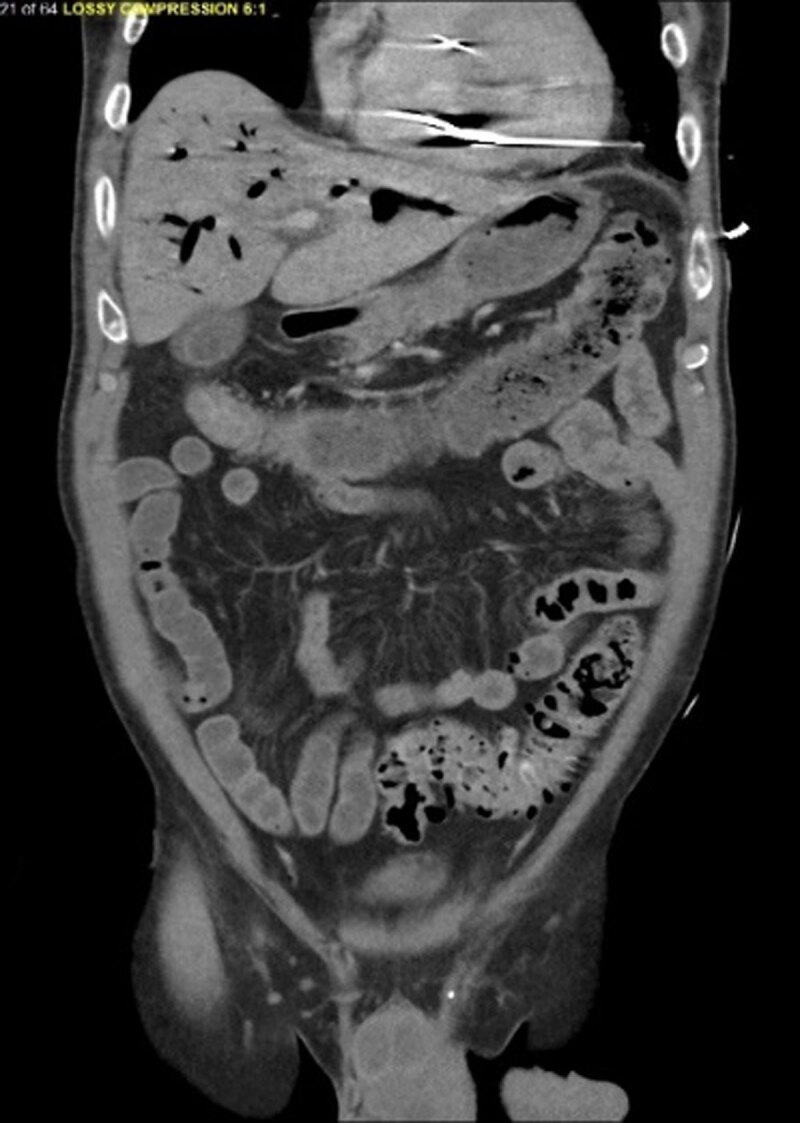
Computed tomography of the abdomen showing a venous air embolism in right ventricle and portal system.

**Figure 5:**
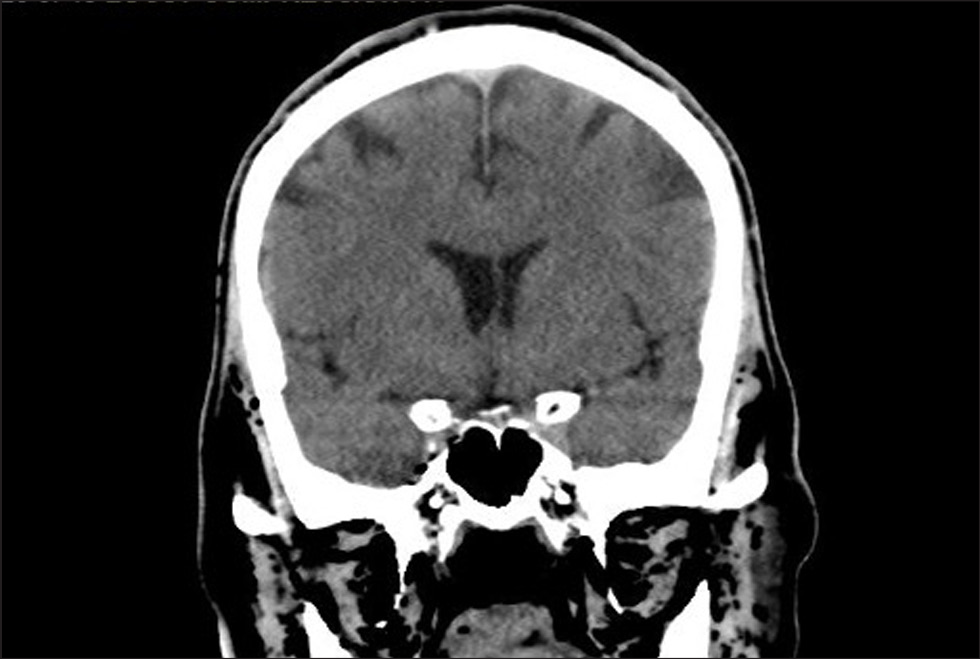
Computed tomography of the head revealing the presence of air in facial tissue.

Electrocardiogram showed sinus tachycardia with right bundle branch block (Figure 1). Computed tomography (CT) of the head demonstrated extensive free air within the maxillary bone, facial tissues, and neck veins, and a thoracic CT showed large amounts of air within the RV and portal venous system (Figures 2, 3, 4, 5). Echocardiogram revealed a left ventricular ejection fraction of 20% to 25%, with right ventricular dilation and reduced function. There were artifacts due to the presence of air in the RV. Initial blood work revealed a leukocyte count of 8,400 cells/μL, hemoglobin of 13.1 g/dL, and a platelet count of 78,000 cells/μL. Moreover, sodium level was 140 mmol/L, potassium 3.6/ mmol/L, magnesium 2.2/ mmol/L, and creatinine 1.23 mg/dL. Patient’s troponin at point of arrival was 0.017 ng/mL (reference range 0.000-0.039) and N-terminal pro-brain natriuretic peptide was 107 pg/mL.

The patient was admitted to the ICU, and supportive care was initiated along with targeted temperature management and VAE-specific interventions, including left lateral decubitus head-down positioning, and high-FiO2 mechanical ventilation. Although VAE significantly improved, he did not have meaningful neurologic recovery and the decision was made to withdraw care.

## Discussion

Subcutaneous emphysema, pneumomediastinum, or VAE can result from spontaneous, traumatic, infectious, or iatrogenic etiology.^6^ During dental procedures, high-speed air turbine drills are utilized to cut a tooth at rotational speeds of 450,000 rpm, while air and water is discharged to cool the heat created by friction at the cutting surface.^6^ The incidence of VAE is also dependent on RV function, volume status, and the presence and extent of pulmonary hypertension. The incidence is higher in patients with hypovolemia or higher RV contractility.^7^

There are multiple perioperative monitoring techniques that can detect venous air embolism, such as transthoracic echocardiogram, transesophageal echocardiogram, end tidal carbon dioxide monitoring, and pulmonary artery catheter that can detect an increase in pulmonary artery pressure.^1^ There are certain measures that can be used to prevent VAE.^1^ The patient should be well hydrated to maintain higher right atrial pressure with a goal of 10 to 15 mm Hg.^1^ Military anti-shock trousers can be utilized on the abdomen to increase right atrial pressure in patients undergoing a procedure in the sitting location.^1^ In patients who are on mechanical ventilation, the use of higher positive end expiratory pressure can reduce the gradient and reduce the incidence of VAE.^1^ It is beneficial to have a lower operative site below the level of the heart, when possible.^1^

Treatment of VAE consists of positioning the patient in a Durant’s maneuver—left lateral decubitus, head down position—which helps to trap the air embolism in the RV.^2,3^ The use of a Swan Ganz catheter or single-lumen central line could be used to aspirate the air.^2^ Moreover, positioning the patient in the Trendelenburg position can decrease the central venous pressure and improve hemodynamics.^2^ Administration of 100% oxygen via a nonrebreather mask can replace air or nitrous oxide with oxygen and hasten the resolution of emphysema.^5,8^ Upon recognition of VAE, the surgical site should be flooded with saline to avoid further air entrainment.^1^

## Conclusion

We present a rare case of fatal VAE that occurred during a routine dental root canal procedure. VAE should be recognized as a potentially fatal complication from routine procedures. In this paper, we review the current literature, discuss the common etiologies and review management options of VAE.
